# The impact of attachment distress on affect-centered mentalization: An experimental study in psychosomatic patients and healthy adults

**DOI:** 10.1371/journal.pone.0195430

**Published:** 2018-04-19

**Authors:** Anna S. Herrmann, Manfred E. Beutel, Katharina Gerzymisch, Richard D. Lane, Janine Pastore-Molitor, Jörg Wiltink, Rüdiger Zwerenz, Mita Banerjee, Claudia Subic-Wrana

**Affiliations:** 1 DFG Research Training Group “Life Sciences, Life Writing” (GRK2015/1) / Department of Psychosomatic Medicine and Psychotherapy, University Medical Center Mainz, Mainz, Germany; 2 Department of Psychosomatic Medicine and Psychotherapy, University Medical Center Mainz, Mainz, Germany; 3 Department of Psychiatry, The University of Arizona College of Medicine, Tucson, Arizona, United States of America; 4 Department of English and Linguistics, University of Mainz, Mainz, Germany; Universite du Quebec a Trois-Rivieres, CANADA

## Abstract

**Introduction:**

We investigated the impact of attachment distress on affect-centered mentalization in a clinical and a non-clinical sample, comparing mentalization in a baseline condition to mentalization under a condition of attachment distress.

**Methods:**

The sample consisted of 127 adults who underwent inpatient psychosomatic treatment, and 34 mentally healthy adults. Affect-centered mentalization was assessed by analyzing participants’ narratives on interpersonal situations in a baseline condition with the Levels of Emotional Awareness Scale (LEAS), and an experimental condition inducing attachment distress with the Adult Attachment Projective Picture System (AAP). Unlike the LEAS, the AAP is specifically designed to trigger attachment distress. In both conditions, the narratives were evaluated using the LEAS scoring system. Additionally, we assessed the impact of childhood trauma on affect-centered mentalization with the Childhood Trauma Questionnaire (CTQ).

**Results:**

While the non-clinical sample displayed the same level of affect-centered mentalization in both conditions, the majority of the clinical sample reached higher scores in the attachment distress condition. There was no strong relationship between reported trauma and mentalization scores.

**Discussion:**

Our findings lend strong empirical support to the assumption that affect-centered mentalization is modulated by attachment-related distress. Several possible explanations for the differences between and within the clinical and the non-clinical sample are discussed.

## Introduction

Since Bowlby’s introduction of attachment theory [1–3], a growing body of literature has provided evidence for a close association between attachment and health. In their groundbreaking review, Maunder and Hunter [4] showed that attachment insecurity can be empirically linked to a wide range of physical diseases, while secure attachment has been found to be associated with physical health. These relationships also hold true for mental health [5]. Maunder and Hunter [4] identified three paths linking adverse early-life experiences and the resulting attachment insecurity with risks for physical and mental well-being: attachment insecurity leads to disturbances in bodily stress regulation systems, increases the use of external stress regulators, and impairs the proper use of support by others.

A key underlying mechanism is the short- and long-term regulatory function of attachment relationships, specifically concerning negative affects [6]. In their social biofeedback model, Gergely and Watson [7] elaborate how the interactional process between an infant and her caregivers shapes emotional self-awareness and the ability to regulate emotions. They emphasize the central role of “marked affect-mirroring”: an infant needs her emotions to be mirrored by her caregiver correctly and in a way that marks that they belong to the infant, not to the caregiver. Apart from contributing to the regulation of an infant’s current state, this is the precondition for her to develop a secondary representation of her own mind, which constitutes the foundation for what Fonagy and colleagues have termed “mentalized affectivity” as part of their larger theory of mentalization [8]. Mentalized affectivity, or affect-centered mentalization, is the ability to identify, reflect on, express, and regulate one’s emotions, and to differentiate between one’s own and another person’s emotional state. Affect-centered mentalization is regarded as highly relevant for health and successful interpersonal interaction [8]. The ability to perceive and regulate negative affect is assumed to be of particular importance to health, as aversive emotional states which cannot be differentiated and regulated are experienced as distressing and may lead to the use of external regulators if functional coping strategies are unavailable (e.g. emotional eating or the use of drugs and alcohol). Unsuccessful management of negative affect may further cause damage in interpersonal relationships.

Empirical evidence largely supports the assumption that affect-centered mentalization is important for physical and mental health. The few studies that have investigated the relationship between affect-centered mentalization and physical health [9–11] all show a positive association. Concerning mental health, studies provide evidence for deficits or alterations of mentalization across a wide range of mental health disorders including borderline personality disorder [12–19], somatoform disorders [20–24], affective and anxiety disorders [18,21,25–33], eating disorders [26,30,34–37], substance use disorders [38–44], post-traumatic stress disorder [45–47], and schizophrenia [48,49].

Caregivers vary in their attachment security and ability for affect-centered mentalization, and thus in their ability to correctly interpret, mirror, and adequately or “contingently” respond to an infant’s affective signals and needs [50]. This has been shown to affect infant attachment [51–56], implicating consequences for the development of affect-centered mentalizing as well. Attachment relationships characterized by traumatic experiences such as emotional, physical, or sexual abuse are assumed to have particularly far-reaching consequences concerning the development of emotional self-awareness and affect regulation: in such cases, the caregiver, needed as a psychological safe haven, is a source of danger at the same time, creating a so-called “fright without solution” situation for the child [57]. As a consequence, the child is less likely to develop accurate secondary representations of her own mind and internalize the experience that aversive emotions are tolerable and can be regulated, resulting in a limited ability for affect-centered mentalization in adulthood. Additionally, she may not learn to safely explore others’ minds, as they have been experienced as potentially threatening [8,58]. Empirical evidence for the postulated theoretical link between childhood trauma and affect-centered mentalizing is still scarce, however.

Considering the rising evidence on the importance of affect-centered mentalization, it is crucial to understand under which circumstances deficits or alterations of affect-centered mentalization manifest themselves. As Fonagy and colleagues have convincingly theorized and empirically demonstrated especially in patients with Borderline Personality Disorder [59], they are most likely to emerge in situations of attachment-related distress, such as being left by an important attachment figure like a partner or close friend. Such distressing, attachment-relevant situations invoke an individual’s “internal working model” of attachment [2]. Functioning largely unconsciously, internal working models guide the interpretation and regulation of emotions, thoughts, and behavior in attachment-relevant situations. Studies by other authors also suggest a close relationship between attachment and mentalization [60–63], but only very few studies have directly investigated the impact of attachment distress on affect-centered mentalization [60,64].

The present study is one of the first studies with a large clinical and a non-clinical sample to investigate the impact of attachment distress on affect-centered mentalization, taking into account the role of childhood trauma. We use an experimental design to compare the ability to mentalize in a baseline condition with the ability to mentalize under a condition of attachment distress in a sample of psychosomatic inpatients with high rates of childhood trauma as well as a sample of mentally healthy adults with no history of childhood trauma. The study is guided by the hypotheses that 1) attachment distress lowers affect-centered mentalization, and that 2) psychosomatic inpatients display a lower level of affect-centered mentalization than mentally healthy adults. In the clinical sample, it further investigates the hypothesis that 3) a history of childhood trauma is associated with a lower level of affect-centered mentalization.

## Methods

### Participants

The sample for this study comprised 127 adults who underwent inpatient treatment at the Department of Psychosomatic Medicine and Psychotherapy at the University Medical Center Mainz, Germany, in between 2007 and 2010. It additionally included a non-clinical sample of 34 adults with no mental health disorders, originally recruited for two other studies conducted at the department [65,66]. The absence of mental health disorders, including personality disorders, in the non-clinical sample had been confirmed using the German version of the Structured Clinical Interview for DSM Disorders (SCID) [67]. The non-clinical participants were further characterized by an absence of traumatic experiences in childhood, defined as not fulfilling the criteria put forward by Walker and colleagues for clinical significance of any type of abuse measured by the Childhood Trauma Questionnaire (CTQ) [68]. In order to be included in the study, all participants were required to complete the CTQ [69], the Levels of Emotional Awareness Scale (LEAS) [70], and the Adult Attachment Projective Picture System (AAP) [71]. The average age of the study participants was 40.1 (SD = 12.9) years in the clinical and 39.2 (SD = 13.4) years in the non-clinical sample. Both samples were characterized by a high proportion of females (70.1% of patients and 64.7% of non-clinical participants), and a relatively high level of education (49.6% of patients and 50.0% of non-clinical participants held at least an advanced high school diploma). The clinical and the non-clinical sample did not differ in age (*t* = .365, *p* = .72), sex (χ^2^ = .362, *p* = .55), or level of education (χ^2^ = .002, *p* = .97). In the clinical sample, the primary ICD-10 diagnosis as obtained from the clinic’s records determined the assignment to one of four diagnostic groups (see Table 1).

**Table 1:** Primary ICD-10 diagnoses in the clinical sample (*n* = 127).

Depressive disorders: F32, F33, F34	*n* = 52 (40.9%)
Anxiety and obsessive-compulsive disorders: F40, F41, F42	*n* = 27 (21.3%)
Somatoform disorders: F45	*n* = 27 (21.3%)
Other disorders: F43, 44, F48, F50, F51, F54, F60, F95	*n* = 21 (16.5%)

Most patients (91.3%) were diagnosed with at least one additional mental health disorder.

### Measures

The patients completed all measures as well as a detailed sociodemographic questionnaire in the week after intake for a psychosomatic treatment program: CTQ, LEAS, and the sociodemographic questionnaire were administered at intake, followed by the AAP one to three days later. The non-clinical participants first completed a short sociodemographic questionnaire and were administered the SCID, the CTQ, and the LEAS. They were invited again for the AAP interview a few days later. In this study, both LEAS and AAP were employed to determine each participant’s ability for affect-centered mentalization, allowing for a comparison of this ability in a baseline condition (LEAS), characterized by the absence of attachment-related distress, versus under the condition of a stepwise induction of attachment distress (AAP). All participants gave informed consent to the use of their anonymized data in studies conducted at the department. In case of the patients, who completed all measures as part of the routine pre-treatment assessment, consent was obtained verbally. The non-clinical participants gave written consent. Ethical approval for this study was given by the ethics committee of the Federal State of Rhineland Palatinate (confirmation letter 01.12.2016 / Wa).

#### Childhood Trauma Questionnaire

The CTQ [69] is a 28-item self-report questionnaire for the retrospective assessment of traumatic experiences in childhood. The CTQ consists of five subscales: emotional, physical, and sexual abuse, emotional and physical neglect. Like the American original, the German version has proven to be an efficient, reliable and valid screening for experiences of abuse and neglect in childhood. The only exception is the physical neglect subscale, which has not shown satisfactory internal consistency [72,73]. The CTQ specifically refers to experiences with one’s family members or primary caretakers. Items are answered on a five-point Likert scale. The lowest possible total score on each subscale is 5, the maximum score is 25. Walker and colleagues have provided cut-off values to determine the clinical significance of each type of abuse or neglect [68]. The CTQ’s primary purpose in this study was to determine the prevalence and degree of traumatic experiences in childhood, and to investigate the relationship between childhood trauma and affect-centered mentalization in the clinical sample. For this purpose, the clinical sample was split using a dichotomous criterion: patients who scored at or above the clinical cut-off on at least one of the three abuse subscales were assigned to a group called “clinically significant abuse”, while the others were assigned to a group called “no clinically significant abuse”. Finally, the CTQ was used in this study to screen the non-clinical participants for the absence of abuse experiences before including them in the study. Applying the Walker cut-off values is a high-threshold criterion for the presence of clinically significant traumatization. It was chosen to enable a highly-targeted investigation of the impact of childhood trauma on affect-centered mentalization, ensuring clinical relevance. For the same reason and because of their superior psychometric properties, only the abuse subscales were taken into consideration.

#### Levels of Emotional Awareness Scale

The LEAS [70,74] is a reliable and valid paper and pencil performance measure developed according to the Levels of Emotional Awareness theory. It is based on an integration of Piaget’s theory of sensory-cognitive development and Werner and Kaplan’s work on symbolization, positing that the psychological capacity to process affect develops along five stages from implicit to explicit processing [75–77]. The LEAS consists of 20 descriptions of interpersonal interactions between a protagonist and another person. Participants are asked to describe in writing how they would feel if they were the protagonist and how they would feel if they were the other person. The answers are rated using the LEAS scoring system, a tool to analyze language with a five-point scale to determine the level of emotional awareness. Higher scores indicate a higher level of emotional awareness: levels 1 and 2 characterize affect processing at an implicit level, such as descriptions of physical sensations or action tendencies, while levels 3 through 5 characterize affect processing at an explicit level. For example, a participant’s description of one single differentiated emotion would be assigned a score of 3, while a score of 5 would be given for a description of two different sets of complex, ambivalent emotions in the protagonist and in the other person. The LEAS manual details the criteria that must be met for each level and includes a glossary that assigns fixed values to the words most commonly used by participants in their answers. Emotional awareness was used to operationalize affect-centered mentalization in this study, and participants’ scores in the LEAS were used to establish their baseline capacity for affect-centered mentalization. Unlike the AAP, the LEAS is not designed to induce attachment distress. The LEAS can be split into two statistically parallel 10-item versions (A and B). Version A was used for this study [74].

#### Adult Attachment Projective Picture System

The AAP [71] is an interview-based assessment of adult attachment. It consists of seven pictures, presented to participants in a predetermined order. Interviewees are asked to describe what is happening in each picture, what led up to the events, what the characters are thinking or feeling, and what will happen next, resulting in a short narrative about each scene. Developed in accordance with attachment theory, the pictures show attachment-related scenes such as solitude, separation, illness, and death, triggering and gradually increasing attachment-related distress. The test’s authors report that the pictures are such powerful stimuli that it is possible for interviewees to become too distressed to continue, or to need a debriefing or a follow-up [71]. Besides extensive clinical observation, results of neuroimaging studies further substantiate the claim that the AAP successfully activates the attachment system at the level of unconscious representations [61,78]. A further study showed that AAP pictures are perceived as significantly more emotionally draining than similar but neutral pictures not depicting attachment-related scenes [60]. Trained student assistants conducted the AAP interviews for this study. AAP interviews are recorded, transcribed, and, when using the AAP toward its original purpose, rated by trained judges in order to determine a participant’s attachment status. In this study, however, the transcripts were instead rated using the LEAS scoring system (see above), in order to determine the level of affect-centered mentalization observable in participants when exposed to attachment distress. In order to apply the LEAS scoring system to AAP narratives, the LEAS manual was adapted to their specific characteristics. The LEAS scoring system has been successfully applied to other types of narratives before [20,22]. Reliability was ensured by conducting an inter-rater reliability study with two independent raters scoring 10 randomly selected AAP transcripts comprising 70 AAP narratives. The resultant intra-class correlation of .87 was considered satisfactory.

### Statistical analysis

The statistical analysis was conducted using SPSS Version 22. Group comparisons were done using *t*-tests for independent samples or ANCOVA if there was a need to control for covariates. Paired *t*-tests were employed for paired comparisons. Comparisons of more than two groups were done using ANOVA or ANCOVA. Correlations were calculated using Pearson’s Product-Moment Correlation. The level of significance was set at *p* < .05.

## Results

### Childhood trauma rates

Table 2 presents the mean scores and the rates of clinically significant childhood trauma on the CTQ abuse scales according to the cut-off values provided by Walker [68]. Overall, 62.2% of patients had experienced at least one type of clinically significant abuse.

**Table 2:** CTQ scores and rates in the clinical sample.

CTQ subscale	Cut-off	*Mean* (*SD*)	% above cut-off
Emotional abuse	≥ 10	11.68 (5.45)	57.5
Physical abuse	≥ 8	7.87 (4.55)	31.5
Sexual abuse	≥ 8	7.18 (5.01)	18.9

*SD* = standard deviation

In the clinical sample, women scored significantly higher than men on the sexual abuse subscale (*M*_Women_ = 8.07, *M*_Men_ = 5.11, *t* = -4.818, *p* < .001). Age was positively correlated with the physical abuse subscale (Pearson’s *r* = .224, *p* = .011). Finally, better-educated participants had significantly lower scores than less educated participants on the physical (*F(2*,*124*) = 6.818, *p* = .002) and the sexual abuse subscale (*F(2*,*124*) = 5.477, *p* = .005). There were no significant differences between the diagnostic groups on any of the CTQ abuse subscales. In the non-clinical sample, there were no differences concerning sex, age, or education.

### Affect-centered mentalization in LEAS and AAP

As Table 3 shows, the patients had a significantly higher affect-centered mentalization score in the attachment distress condition than in the baseline condition, while no such difference was found in the non-clinical sample. The non-clinical sample reached a significantly higher affect-centered mentalization score at baseline than the clinical sample, while there was no difference between the samples in the attachment distress condition (see Table 3).

**Table 3:** Affect-centered mentalization scores in the clinical vs. the non-clinical sample, and in the baseline (*M*_LEAS_) vs. the attachment distress condition (*M*_AAP_).

Sample		*M*_LEAS_ (*SD*)	*M*_AAP_ (*SD*)	Paired *t*-tests	
				*t*-value	*p*-value
Clinical (*n* = 127)		2.75 (.54)	3.18 (.47)	-8.417	< .001
Non-clinical (*n* = 34)		3.21 (.56)	3.21 (.43)	-.052	.96
***t*-test for indepen-dent samples**	*t*-value	-4.357	-.353		
	*p*-value	< .001	.73		

*M*_LEAS_ = mean affect-centered mentalization score in the LEAS (baseline condition), *M*_AAP_ = mean affect-centered mentalization score in the AAP (attachment distress condition), *SD* = standard deviation

**Fig 1:**
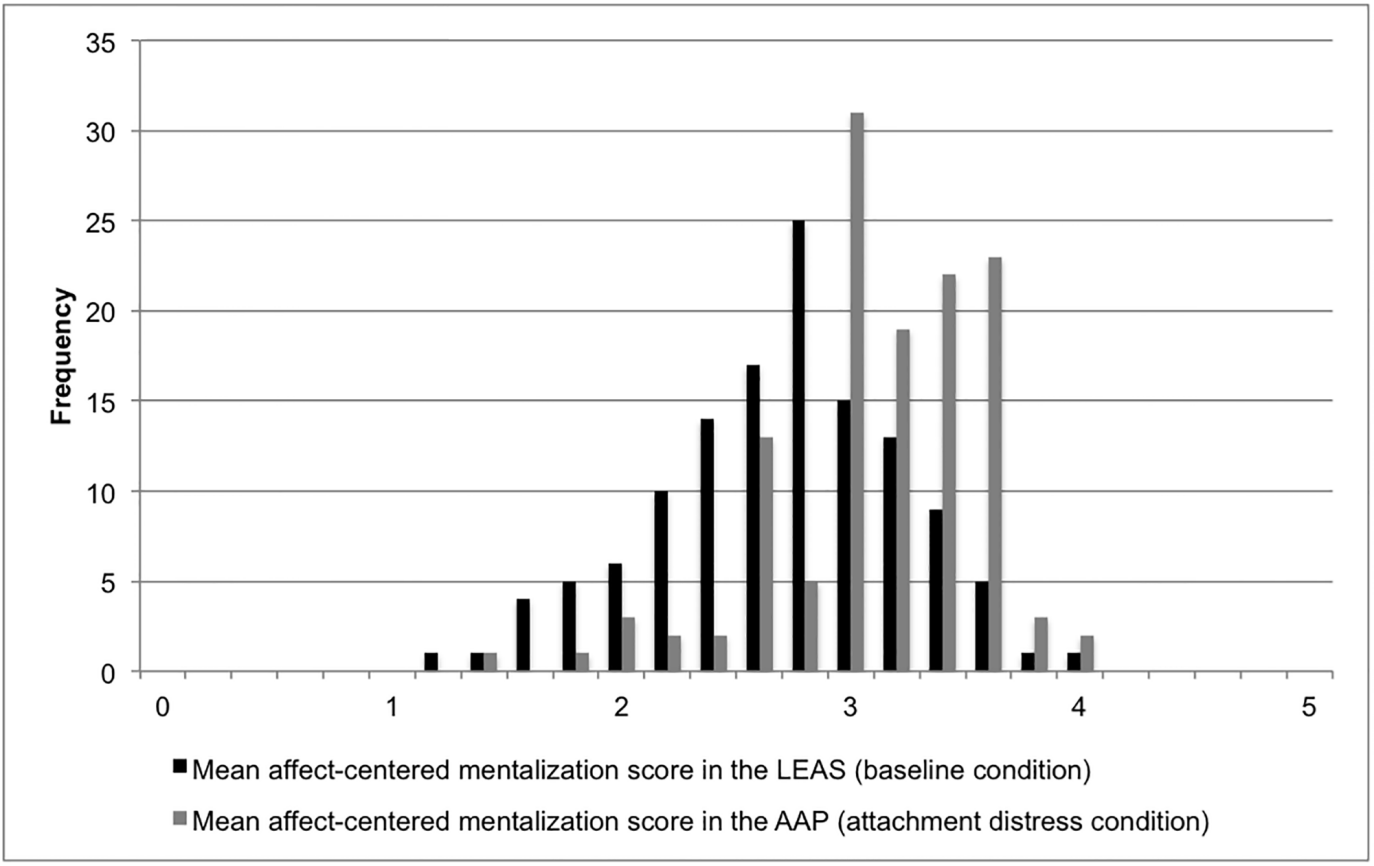
Affect-centered mentalization scores in the baseline and the attachment distress condition in the clinical sample (*n* = 127).

**Fig 2:**
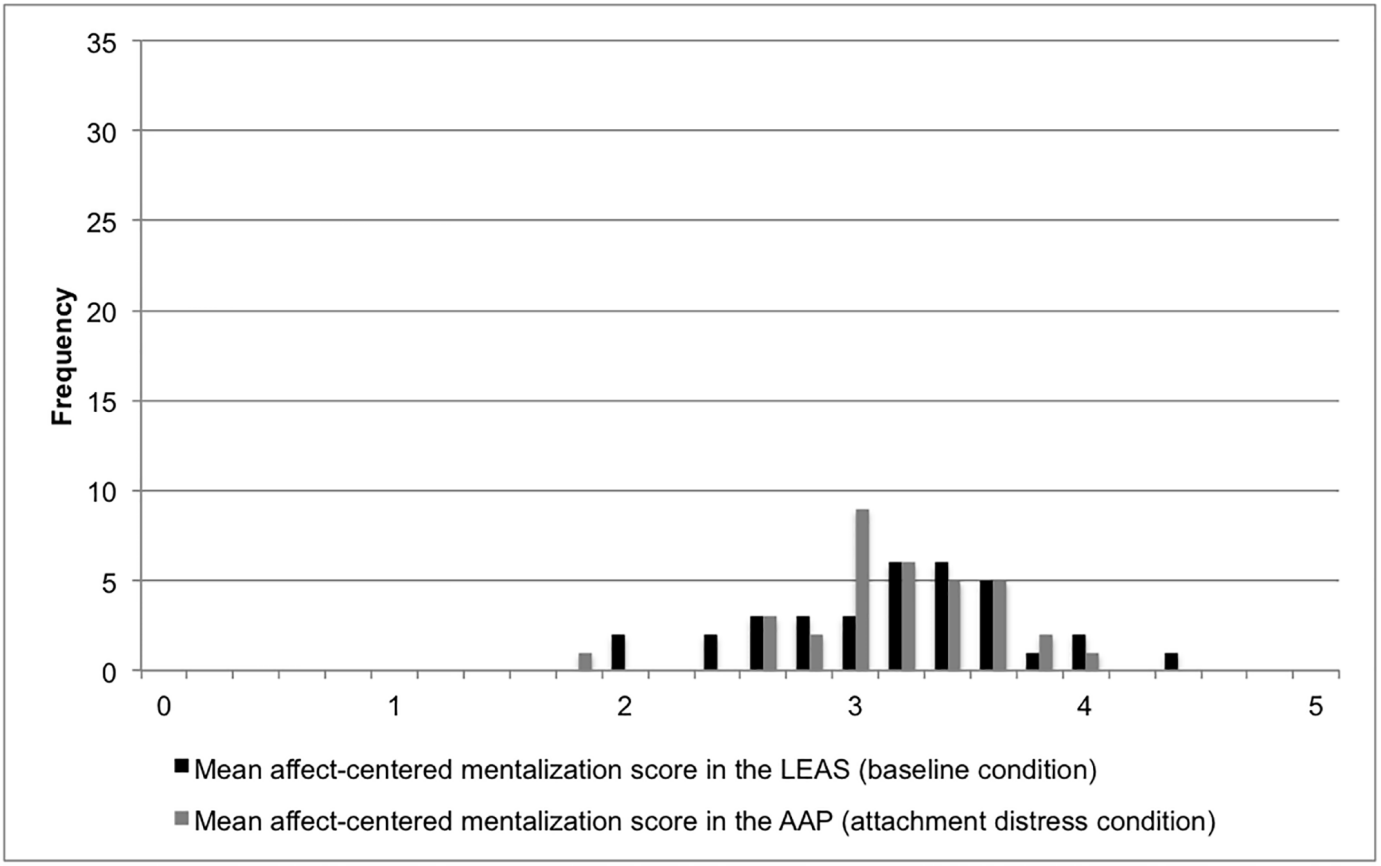
Affect-centered mentalization scores in the baseline and the attachment distress condition in the non-clinical sample (*n* = 34).

In the clinical sample, women had a higher affect-centered mentalization score than men in both LEAS (*M*_Women_ = 2.85, *M*_Men_ = 2.51) and AAP (*M*_Women_ = 3.22, *M*_Men_ = 3.11), a sex difference only significant in the LEAS (*t* = -3.386, *p* = .001). The same held true for the non-clinical sample (*M*_Women_ = 3.37, *M*_Men_ = 2.92, *t* = -2.385, *p* = .023). Age was negatively correlated with mentalization to a significant degree only in the clinical sample in both LEAS (*r* = -.306, *p* < .001) and AAP (*r* = -.201, *p* = .023). Better-educated participants had significantly higher mentalization scores than less educated participants only in the clinical sample, and only in the LEAS (*F(2*,*124*) = 6.525, *p* = .002). No significant differences between the diagnostic groups were found in either condition. Figs 1 and 2 illustrate the distribution of scores in the baseline and the attachment distress condition in the clinical and in the non-clinical sample.

To enable a more detailed analysis of the difference between the affect-centered mentalization scores in the baseline and the experimental condition in the clinical sample, a difference score between the baseline and the attachment distress condition was created by subtracting the affect-centered mentalization score in the AAP from the affect-centered mentalization score in the LEAS. The mean difference was -.43 (*SD* = .58), with individual differences ranging from -2.03 to 1.26. A majority of 80.3% (*n* = 102) of patients reached higher affect-centered mentalization scores in the attachment distress condition than at baseline, while a minority of 19.7% (*n* = 25) reached lower affect-centered mentalization scores in the attachment distress condition than at baseline. In 49.6% (*n* = 63) of cases, the difference between the affect-centered mentalization score at baseline and under attachment distress was at least .5.

### The impact of trauma in the clinical sample

In order to investigate the relationship between childhood trauma and affect-centered mentalization, participants’ CTQ scores on each abuse subscale were correlated with their affect-centered mentalization scores in the baseline and in the attachment distress condition. Emotional abuse was not significantly correlated with affect-centered mentalization in the baseline (*r* = -.088, *p* = .325) or the attachment distress condition (*r* = -.022, *p* = .810). Physical abuse was significantly, negatively correlated with affect-centered mentalization in the baseline (*r* = -.185, *p* = .037), but not in the attachment distress condition (*r* = -.004, *p* = .963). Sexual abuse was not significantly correlated with affect-centered mentalization in the baseline (*r* = -.002, *p* = .985) or the attachment distress condition (*r* = .033, *p* = .709). Additionally, the clinical sample was divided into a “clinically significant abuse” and a “no clinically significant abuse” group, the criterion being a score at or above the clinical cut-off [68] on at least one of the three CTQ abuse subscales. The two groups’ affect-centered mentalization scores at baseline and under attachment distress as well as their difference scores were compared. Controlling for sex, age, and education by ANCOVA, there were no significant differences between the groups (see Table 4).

**Table 4:** Affect-centered mentalization scores in the baseline (*M*_LEAS_) and in the attachment distress condition (*M*_AAP_) and difference score (*M*_LEAS-AAP_) of clinically significant abused vs. not abused participants.

	Clinically significant abuse (*n* = 48)	No clinically significant abuse (*n* = 79)	ANCOVA	
			*F*-value	*p*-value
*M*_LEAS_ (*SD*)	2.77 (.57)	2.74 (.52)	.058	.81
*M*_AAP_ (*SD*)	3.19 (.48)	3.18 (.43)	.003	.96
*M*_LEAS-AAP_ (*SD*)	-.42 (.57)	-.44 (.59)	.026	.87

*M*_LEAS_ = mean affect-centered mentalization score in the LEAS (baseline condition), *M*_AAP_ = mean affect-centered mentalization score in the AAP (attachment distress condition), *M*_LEAS-AAP_ = mean difference score (score in the AAP subtracted from score in the LEAS), *SD* = standard deviation, ANCOVA = analysis of covariance (control variables: sex, age, and education)

Finally, a comparison between the patients who improved and the patients who deteriorated in their capacity for affect-centered mentalization when exposed to attachment distress, controlling for sex, age, and education by ANCOVA, did not yield differences between their CTQ scores (see Table 5).

**Table 5:** CTQ scores of patients who improved and deteriorated in the attachment distress condition.

	Improved (*n* = 102)	Deteriorated (*n* = 25)	ANCOVA	
			*F-value*	*p-value*
Emotional abuse *Mean (SD)*	11.80 (5.43)	11.18 (5.61)	.733	.394
Physical abuse *Mean (SD)*	8.11 (4.85)	6.91 (2.85)	2.051	.155
Sexual abuse *Mean (SD)*	7.20 (5.13)	7.11 (4.63)	.557	.457

*SD* = standard deviation, ANCOVA = analysis of covariance (control variables: sex, age, and education)

## Discussion

This study aimed to investigate the impact of attachment distress on affect-centered mentalization in psychosomatic inpatients and mentally healthy adults. It compared the ability for affect-centered mentalization in a baseline condition, characterized by the absence of attachment distress, with the ability for affect-centered mentalization under a condition of attachment distress, induced by an instrument that activates the attachment system at the level of unconscious representations. In the clinical sample, the study additionally investigated the impact of a history of childhood trauma on affect-centered mentalization in both conditions. In a nutshell, the results show affect-centered mentalization to increase under attachment distress, but only in the clinical sample, which was characterized by a lower baseline level of affect-centered mentalization than the non-clinical sample. There was no strong relationship between reported trauma and affect-centered mentalization scores.

When applying the LEAS scoring system to LEAS narratives, produced in response to fictitious scenarios not designed to induce attachment distress, the resulting distribution of affect-centered mentalization in the clinical sample approaches a normal distribution with a mean score of 2.75 (SD = .54). When applying the same measure for affect-centered mentalization to AAP transcripts and thus to narratives produced under a condition of attachment distress, a different picture emerges: the distribution is narrower and slightly left-skewed, with a mean score of 3.18 (SD = .47). In the five-level LEAS scoring system, this improvement indicates a switch from implicit mentalizing to explicit mentalizing. Under attachment distress, patients show a distinctly different pattern of affect-centered mentalization than at baseline, with almost half of them (49.6%) differing by at least .5 or “half a level” of emotional awareness between conditions. Patients’ individual differences between conditions do not all follow the same pattern, however, ranging from an improvement in affect-centered mentalizing under attachment distress of up to 2.03, to a deterioration of affect-centered mentalizing under attachment distress of up to 1.26. This reflects a wide variation of reactions to attachment distress, with the clear majority (80.3%) of patients improving their capacity for affect-centered mentalization—the opposite of our expectation.

A different picture emerges in case of the non-clinical sample, regarding both their baseline ability for affect-centered mentalization and their behavior under attachment distress: with a mean score of 3.21 (SD = .56), the non-clinical sample displays a significantly better baseline capacity for affect-centered mentalization than the clinical sample, mentalizing explicitly. The non-clinical sample further differs from the clinical sample in displaying the same level of affect-centered mentalization in the attachment distress condition (*M* = 3.21, SD = .43) as in the baseline condition. Their ability for affect-centered mentalization seems largely unaffected by an induction of attachment distress. As a result, the clinical and the non-clinical sample mentalize at a very similar level in the attachment distress condition. In both samples and in both conditions, women reached higher affect-centered mentalization scores, albeit the sexes differed significantly only at baseline. In the clinical sample, higher baseline scores were further associated with a younger age and a higher education. These results are in line with previous studies [74,79]. How can these patterns be interpreted?

First, it must be noted that the results speak in favor of the validity of the chosen method. This study broke new ground in adapting the LEAS scoring system to AAP narratives. Using two different tests to operationalize affect-centered mentalization carried the risk that one of the two tests would simply enable affect-centered mentalization better than the other. The fact that not all participants exhibited the same direction or degree of change between conditions makes that possibility seem unlikely.

The marked difference between the two samples at baseline, who were not matched in size but did not differ from each other in sex, age, or level of education, aligns with previous research using the LEAS that non-clinical samples display a better capacity for affect-centered mentalization than clinical samples [15,22,24,26,27,31,36,45]. Based on a large and heterogeneous sample, this study adds strong support to this finding. Interestingly, there were no differences between the diagnostic groups (in either the baseline or the attachment distress condition), a finding which differs from previous studies. For example, Subic-Wrana and colleagues [21] found that patients with somatoform disorders scored significantly lower on the LEAS than patients with other mental health disorders—which, as the authors convincingly argue, is concordant with the assumption that the somatic, rather than symbolic, representation of affect is characteristic for somatoform disorders. The lack of differences between the diagnostic groups in the study at hand may be due to the fact that the great majority (91.3%) of patients in this study had at least one other diagnosis, possibly blurring the boundaries between the groups. It is further possible that the nature of the relationship between affect-centered mentalization and mental illness varies across disorders in a way that is not captured well by the LEAS scoring system. The LEAS scoring system rates differentiation rather than accuracy (or the question: are the feelings ascribed to a LEAS scenario by a participant in accordance with the feelings most people would ascribe to it?). Differences between diagnostic groups concerning accuracy rather than differentiation are therefore hard to detect with it.

The second difference between the clinical and the non-clinical sample concerns their change in performance between the two conditions, suggesting that clinical and non-clinical populations do not only differ in their general capacity for affect-centered mentalization, but also react differently to the induction of attachment distress. The search for an explanation for this difference may be aided by a closer look at the wide variation of change in the clinical sample. The fact that a majority of patients [80.3%] improved when under attachment distress seems surprising at first: generally, attachment distress has been argued to be more likely to have an inhibitory effect on mentalizing [59,80]. As Liotti and Gilbert [80] summarize, “it is possible that the activation of the attachment system, in the face of threats, inhibits mentalizing abilities because the evolutionarily older threat-defence (fight–flight) system (…) normally inhibits higher order cognitive processes” (p. 10). Our findings are, however, compatible with the biobehavioral switch model proposed by Fonagy and Luyten [59] and based on Mayes’s [81,82] work on arousal regulatory neural systems. It posits an effect of interpersonal stress on mentalization dependent on the degree of emotional arousal in the shape of an inverted U-curve: rising stress leads to an increase in mentalization up to a “switch point” at which arousal becomes so high that it has the opposite effect, inhibiting mentalization and instead giving rise to pre-mentalizing modes of functioning. This switch is accompanied by a shift from flexible, controlled neural processes associated with the prefrontal cortex, to automatic processes associated with posterior cortical and subcortical structures. It is thus imaginable that the improvement in affect-centered mentalizing displayed by most patients in the attachment distress condition reflects a response to heightened emotional arousal, but not one that surpassed their individual “switch points”, which would have resulted in a deterioration of affect-centered mentalizing instead. After all, situations triggering attachment distress are the ones most urgently requiring affect-centered mentalizing as it enables the regulation and successful communication of affects. It therefore seems plausible that under attachment distress, people generally increase their efforts and try to maximize their ability to think about themselves and others in terms of mental states, which may even result in a change from implicit to explicit mentalization. This seems to have been the case for the majority of participants in the clinical sample. The fact that attachment distress was induced in an obvious psychological testing environment as opposed to in a “real life situation” may have contributed to the fact that for most patients, it did not increase so much that it would lead instead to a decline in mentalization.

The non-clinical sample, on the other hand, may not have needed to push their abilities, as they were already mentalizing at an explicit level under regular conditions. They may not have experienced an increase in emotional arousal high enough to necessitate a further increase in affect-centered mentalization in order to be able to regulate themselves. These considerations also correspond to Lane and colleagues’ hypothesis that the relationship between emotional arousal and the ability to mentalize affects is quadratic rather than linear [83,84]. A decline in affect-centered mentalization in response to attachment distress was the case for only a minority of patients. This raises the question of what determines the ability to improve or at least maintain the capacity to mentalize one’s own and others’ affects in situations characterized by attachment distress, in contrast to a decrease or even break-down in affect-centered mentalizing.

It seems that a variety of factors may determine individual reactions, including both genetic and developmental influences—especially exposure to childhood trauma, assumed to lower the threshold for the switch [59]. In this study, however, a history of childhood trauma was not found to be associated with affect-centered mentalization in the baseline or the attachment distress condition. This may simply be due to the lack of variance in the sample concerning trauma: even though 37.8% of patients did not meet the criteria for clinically significant abuse, they may still have been exposed to more traumatic experiences in childhood than the general population. It seems more likely, however, that the relationship between childhood trauma and affect-centered mentalization is so complex that it does not necessarily present itself in a direct, clear-cut association: Ensink and colleagues [85], for example, did not find a history of childhood trauma to lead to general, but to content-specific mentalization deficiencies, noting that this “raises questions regarding the pathways through which adults who have been abused as children acquire mental state thinking and suggests that some individuals find opportunities, possibly outside the family, to develop a mental state understanding as a way of surviving challenging emotional experiences” (p. 9]. This may be true for some of the patients with a history of childhood trauma in this study: some may have had access to another trusted adult to help them mentalize their experience, limiting the damage these experiences may have had on the development of their mentalization skills and resulting in a reduced likelihood that their ability to mentalize breaks down under attachment distress. Fonagy and Luyten [59] similarly argue that whether or not an individual’s early social environment facilitated a coherent, reflective discourse about mental states may be just as or even more important to the development of mentalization than the fact of maltreatment itself—as do the authors of two empirical studies who also did not find an association between childhood trauma and mentalization: Subic-Wrana and colleagues [86] did not find a linear relationship between CTQ scores and LEAS scores in their sample of psychotherapy inpatients, and Stacks and colleagues [87] did not find reflective functioning to be associated with experiences of severe child maltreatment in a sample of mothers. These findings indicate that it is possible for affect-centered mentalization to unfold in spite of traumatic experiences in childhood—or, put another way, that childhood trauma is only one among many risk factors for the development of deficits in affect-centered mentalizing. Encouragingly, there is evidence that in cases where the ability to mentalize could unfold in spite of traumatic experiences in childhood, it may also act as a buffer against their destructive potential with regard to mental health and social functioning in adulthood [88,89]. Another reason for the lack of a clear association between childhood trauma and affect-centered mentalization found in this study could lie in the observation that in some instances, traumatized individuals may even be particularly sensitive toward mental states [13,14,16,17]. This phenomenon has been discussed as a result of the constant vigilance sometimes adopted by abuse victims as a protective measure and may have shown itself in this study as well.

Returning to the differing reactions to attachment distress in the clinical and the non-clinical sample, another possible explanation is that at least in some cases of apparent improvement in the attachment distress condition in the clinical sample, patients’ higher scores do not reflect functional affect-centered mentalizing, but a “pseudo-mentalizing” mode, such as increased or “hyper-mentalizing” which does not authentically reflect or lead to a successful regulation of affect [90]. The difference between a mentalizing and a pseudo-mentalizing mode may be difficult to detect with the LEAS scoring system. Whether an individual increases, decreases, or reverts to a pseudo-mentalizing mode in their affect-centered mentalizing in response to attachment distress may be modulated by attachment style: Taubner and colleagues [63] found that only persons characterized by high attachment anxiety deteriorated in their ability to mentalize when reminded of a negative attachment experience. Fizke and colleagues [60] also report that the effect of an activation of the attachment system on emotion reading ability differed depending on attachment style. As it was not possible to include participants’ attachment style as a variable in this study, however, it remains an open question whether attachment style influenced the participants’ reactions to attachment distress.

Finally, we cannot rule out the possibility that the participants in the clinical sample simply found the AAP more engaging than the LEAS, while the participants in the non-clinical sample found them equally engaging. Having been administered the LEAS as part of a relatively lengthy battery of pre-treatment tests, the patients may not have been as motivated for the LEAS as the non-clinical participants, while the AAP was conducted as a separate interview (a more engaging task in itself) in both groups.

Affect-centered mentalization is a capacity assumed to be highly relevant for health and successful interpersonal interaction [8]. This is especially true in situations of attachment distress, which is why it is so important to understand whether and how affect-centered mentalization is affected by it. This study demonstrated that attachment distress has an impact affect-centered mentalization, but only in a clinical sample and contrary to what had been expected: in reaction to attachment distress, most patients improved their ability to mentalize, and only a minority deteriorated. The non-clinical sample’s ability to mentalize remained the same. This lays the groundwork for future investigations of the question what determines the ability to maintain the ability to mentalize under attachment distress, which has important clinical implications. As Subic-Wrana and colleagues [91] stress, psychotherapeutic success requires the facilitation of affect-centered mentalizing by “the activation of emotional experiencing and of the cognitive processing of this experience by symbolizing and reflecting on the emotions in order to give them meaning” in the therapy session (p. 219). In order to best strike the balance between emotional experiencing and cognitive processing, it is useful to know as much as possible which factors and circumstances may enable or hinder affect-centered mentalization. This study makes clear that a history of childhood trauma is among those deserving further attention in research. It would further be illuminating to investigate this question with regard to specific psychopathologies.

This is the first study to have successfully tested the application of a well-established instrument for the assessment of emotional awareness to narratives elicited by the AAP, constituting a novel methodological approach of high practical value. Limitations of the study are the unequal sample sizes, the fact that we cannot exclude the possibility that the LEAS also has some potential to induce attachment distress, the well-known limitations of the validity of retrospective reports of adverse experiences in childhood [92], the lack of a more differentiated investigation of different trauma types including neglect, and the regrettable circumstance that it was not possible to include attachment style as a variable. Finally, choosing to apply a rating method to material that could also be approached differently might be criticized as a reductionist approach that does not do justice to a construct as complex as affect-centered mentalization. However, the ability to symbolize inner states through language lies at the very heart of the construct, and that is precisely what the LEAS theory and scoring system capture [75,93]. Further strengths lie in the study’s experimental design and its comparatively large and heterogeneous sample.

## Supporting information

S1 Dataset Attachment Distress Mentalization (SAV)
